# Selective GPR17 antagonism enhances structural and functional recovery in animal models of demyelination

**DOI:** 10.1371/journal.pone.0354525

**Published:** 2026-07-27

**Authors:** Dille De Herdt, Evy Lefevere, Véronique Brouwers, Line Hartvig, Emiel Geeraerts, Cheng-Chih Hsiao, J. Q. Alida Chen, Rui Pinto, Guillaume Duvey, Stephen Burbidge, Anja Harmeier, Irene Knuesel

**Affiliations:** 1 Rewind Therapeutics NV, Leuven (Heverlee), Belgium; 2 Excelra, Ghent, Belgium; 3 Neuroimmunology Research Group, Netherlands Institute for Neuroscience, Amsterdam, Netherlands; 4 Department of Experimental Immunology, Amsterdam Institute for Immunology and Infectious Diseases, Amsterdam University Medical Center, Amsterdam, Netherlands; Cleveland Clinic, UNITED STATES OF AMERICA

## Abstract

Myelination, driven by differentiation of oligodendrocyte precursor cells, is critical for metabolic and structural support and efficient axonal signal transmission in neurons. Loss of myelin is a hallmark of multiple sclerosis and other devastating demyelinating disorders. As demyelination persists, neurons become increasingly vulnerable, leading to neurodegeneration and chronic disability. Restoring myelin through endogenous repair mechanisms offers a promising therapeutic approach to mitigate progressive neuronal loss. One key regulator of myelination is the G protein-coupled receptor 17, GPR17, whose chronic upregulation in oligodendrocyte precursor cells is commonly seen with myelin injury. In line with single-nucleus transcriptomic data showing predominant expression of GPR17 in committed oligodendrocyte precursor cells, our postmortem immunohistochemical analyses of MS patient tissue revealed a significant upregulation of GPR17^+^/BCAS1^+^ oligodendrocyte precursor cells adjacent to and in demyelinated lesions. Importantly, remyelinated lesions lacked GPR17 immunoreactivity, consistent with a model in which sustained GPR17 expression is associated with demyelination and impaired oligodendrocyte precursor cell differentiation. To test the impact of pharmacological GPR17 inhibition on remyelination, we evaluated the effects of a novel, selective GPR17 antagonist in cuprizone-induced murine demyelination models. This toxin-induced approach has been widely used to study mechanisms of de- and remyelination, in the absence of the full inflammatory complexity of demyelinating diseases such as multiple sclerosis. We show that oral treatment results in robust functional recovery consistent with remyelination, as evidenced by improved spatial memory and recovery of visual evoked potential latency delays. GPR17 antagonism also accelerated structural remyelination in the corpus callosum and optic nerve. Together, these findings support a role for pharmacological GPR17 antagonism in promoting remyelination and highlight this G protein-coupled receptor as a promising therapeutic target for demyelinating disorders.

## Introduction

Myelination, the process by which oligodendrocytes ensheathe axons with myelin, is essential for efficient nerve conduction and metabolic support of neurons in the central nervous system (CNS). Disruption of this process, or demyelination, is a defining feature of several neurological disorders, including multiple sclerosis (MS), traumatic brain injury, and ischemic or autoimmune demyelinating conditions affecting the CNS. Loss of the protective myelin sheath impairs electrical signal transmission and compromises oligodendrocyte-mediated support, ultimately contributing to the neurological deficits associated with these conditions [1]. Although the CNS possesses some capacity for endogenous myelin repair, this process is negatively influenced by numerous factors, including inflammation, axonal damage, age, and inhibitory cues in the lesion environment [2]. While certain lesions – particularly smaller or early ones – can undergo substantial or even complete remyelination [3], these repair processes are often insufficient or become progressively less effective in chronic disease settings. Persistent demyelination – often linked to central inflammatory processes – and failed repair are strongly associated with neurodegeneration and progressive functional decline across CNS disorders [4–6]. Despite recent advances in immunomodulatory therapies, including approaches targeting central inflammation (e.g., BTK inhibitors [7,8]), disease progression and disability accumulation frequently persist. These observations highlight the need for therapeutic strategies that not only modulate both peripheral and central inflammatory processes (by targeting brain myeloid cells) but also promote efficient and sustained remyelination. This is particularly relevant for patients with progressive MS, where treatment options remain largely limited to symptomatic management. Among emerging targets, G protein-coupled receptor (GPCR) 17 (GPR17) has been identified as a key molecular regulator of oligodendrocyte differentiation and myelination [9]. Under physiological conditions, GPR17 expression is tightly regulated and specifically expressed by a subset of early bipolar NG2 (Neural/glial antigen 2)-positive oligodendrocyte precursor cells (OPCs) in the intact brain and spinal cord [10,11]. However, injury to the myelin sheath is invariably characterized by abnormal and persistent upregulation of GPR17 in OPCs, thereby impeding the final stages of oligodendrocyte maturation and maintaining OPCs in an immature state, as shown in animal models of demyelination, such as experimental autoimmune encephalomyelitis (EAE) and cuprizone- or LPC-induced demyelinating lesions [11–15]. Dysregulated GPR17 has also been described in other CNS pathologies, such as brain ischemia, traumatic brain injury and spinal cord injury, Alzheimer’s disease, ischemic stroke [10,12,13,16–19], as well as in human ischemic lesions [20] and demyelinating lesions [21]. Early reports suggested that GPR17 agonism may induce OPC differentiation [22]; however, more recent studies using more selective pharmacological approaches indicate that antagonism and inhibition of GPR17 functions accelerates OPC maturation and remyelination. Consequently, the prevailing view in the literature is that GPR17 signaling inhibits OPC differentiation.

Genetic and pharmacological studies support the importance of finely regulating GPR17 activity: overexpression leads to severe myelination deficits, while GPR17 knockout accelerates myelination without adverse effects [12]. Early pharmacological approaches to inhibit GPR17 functions enhanced OPC differentiation and remyelination but lacked specificity [12,23–27] leaving open the possibility that the observed effects may involve other receptor targets. In addition to lacking specificity, the mode of action of these modulators also remains to be further characterized, as the identity of the endogenous ligands for GPR17 is still under debate, a factor that has hampered the identification and characterization of selective GPR17 antagonists. Previous studies have suggested that GPR17 might be activated by cysteinyl leukotrienes [18], by purines [28,29], oxysterols such as the brain cholesterol 24(S)-hydroxycholesterol (24S-HC) [30], or most recently, by the cytokine C1q [31]. However, reproducibility efforts have failed to confirm the published data on the activity of cysteinyl leukotrienes, purines and 24S-HC [32,33], and independent validation of C1q is still lacking. Accordingly, GPR17 remains classified as an orphan receptor. Several studies revealed antagonistic activities of GPR17 targeting compounds by using the synthetic small molecule ligand, MDL-29951 demonstrating the modulation of intracellular cAMP levels, primarily via G_ai_ signaling [27,34,35]. Notably, MDL-29,951 is the most selective and specific GPR17 agonist identified to date, and its availability has enabled the identification and characterization of GPR17 antagonists. MDL-29951 was also included in this study, revealing a competitive antagonism of RWT020016.

In early efforts to study GPR17 functions, non-selective GPR17 antagonists such as Pranlukast and Montelukast were used, providing preliminary insights into its role in remyelination. However, their lack of selectivity and specificity limited the ability to directly attribute observed effects to GPR17 antagonism [24,26,27,36]. Nevertheless, these preclinical studies highlighted the therapeutic potential of promoting remyelination, although translating such strategies to clinical benefit has remained challenging. One major challenge is the direct translation of preclinical findings in animal models to complex neurological diseases in humans. While toxin-induced demyelination models, such as the cuprizone paradigm, are well suited to study mechanisms of de- and remyelination in a controlled setting, they do not fully recapitulate the inflammatory and heterogeneous pathology of multiple sclerosis, which must be considered when translating preclinical findings to human disease.

Early clinical trials have demonstrated that remyelination in humans is possible [37,38], yet achieving consistent and clinically meaningful outcomes has proven difficult. In addition to methodological challenges, such as selecting sensitive imaging and biomarker endpoints, past trials have encountered limitations including off-target side effects (e.g., fatigue and insomnia with histamine H3 antagonists such as Clemastine and GSK239512) [39], as well as metabolic disturbances with Bexarotene [40]. Questions have also been raised regarding trial design, including suitability of comparators (e.g., interferon-beta in anti-LINGO-1 trials), which may not have adequately controlled the inflammatory processes. Furthermore, therapeutic modalities involving antibodies (e.g., Elezanumab targeting RGMa) may have been limited by insufficient brain exposure, potentially reducing their efficacy in promoting remyelination [41,42]. While these challenges need to be addressed in future clinical trials, targeting selectively and specifically GPR17 offers a promising therapeutic approach as it shows an almost exclusive expression in the CNS, in OPCs.

In the current study, we investigated a novel, highly potent and selective small molecule GPR17 antagonist RWT020016. This compound was designed to overcome the shortcomings of earlier pharmacological agents by directly promoting endogenous OPC maturation. This study evaluated the selective GPR17 antagonist RWT020016 for its ability to promote OPC differentiation by releasing the injury-induced block at the committed OPC (COP) stage, expected to facilitate remyelination in a pathological context where GPR17 is dysregulated. GPR17 expression was characterized in postmortem human brain and optic nerve tissue, complemented by a bioinformatics approach using single-nucleus RNA sequencing data. In addition, efficacy was examined in toxin-induced demyelination mouse models, focusing on both acute and chronic cuprizone treatment effects, using functional and behavioral readouts – including visual evoked potentials (VEPs) and a Y-maze test assessing spatial working memory – as well as structural readouts, including quantification of myelin basic protein (MBP) expression in the corpus callosum and assessment of myelin ultrastructure via transmission electron microscopy (TEM) in the optic nerve. These findings support the potential of GPR17 antagonism as a transformative therapy for demyelinating diseases.

## Methods

### Single nucleus transcriptome meta-analysis

To generate a comprehensive overview of oligodendrocyte maturation, five public single-nucleus RNA sequencing datasets spanning a range of diseases and subject ages were collected [5,43–46]. Raw count matrices were obtained from their respective repositories, yielding a total of 214,567 nuclei across all dataset. A consistent quality control (QC) and preprocessing pipeline was applied across datasets using the Seurat R package (version 3.1) [47–49] to ensure comparability and minimize technical variability. Cells were filtered based on standard QC metrics, excluding low-quality nuclei characterized by low gene counts or high mitochondrial transcript content, thereby reducing technical noise and potential batch-specific artifacts. To account for dataset heterogeneity and mitigate batch effects arising from differences in sample origin, sequencing platform, or processing pipelines, datasets were integrated using the Seurat integration workflow. This approach aligns shared biological features across datasets while preserving biologically meaningful variation. Following integration, dimensionality reduction was performed using principal component analysis (PCA), with the number of variable features set to 7,500 and 75 principal components retained for downstream analysis. Cells were then embedded using Uniform Manifold Approximation and Projection (UMAP) based on the integrated PCA space. Clustering was performed using a graph-based approach (FindNeighbors and FindClusters functions), with resolution parameters selected to achieve biologically meaningful separation of major cell populations while avoiding overclustering. Cell-type annotation was initially guided by author-provided labels when available and further refined using canonical marker genes for oligodendrocyte lineage populations, including OPCs, committed oligodendrocyte precursors (COPs), and mature oligodendrocytes. Cells belonging to the oligodendrocyte lineage were subsequently subsetted, and integration, dimensionality reduction, and clustering steps were repeated to refine the representation of oligodendrocyte differentiation, resulting in a final dataset of 71,623 cells. Marker genes for each cluster were identified using Seurat’s FindAllMarkers function with the Wilcoxon rank-sum test. Differential expression analyses between selected clusters (e.g., OPC versus COP populations) were performed using FindMarkers with the same statistical framework.

### Human postmortem immunohistochemical analysis

#### Sample acquisition.

Human brain tissues were provided by the Netherlands Brain Bank (NBB, Amsterdam, The Netherlands; https://www.brainbank.nl). Samples were accessed between 01.01.1996 and 31.12.2018. Use of brain autoptic samples and procedures was approved by the Ethics Committee of Amsterdam UMC (Location VUmc, Amsterdam, The Netherlands), an Institutional Review Board registered with the U.S. Office of Human Research Protections (IRB number: IRB00002991; Federal-wide Assurance number: 00003703). The ethical declaration of the NBB and the Letter of approval of the VU University Medical Center are accessible here: Ethics | Netherlands Brain Bank. All analyses were conducted at the NBB, who also provided the pertinent anonymized donors’ clinical records. Cerebral tissues and optic nerves from eight donors diagnosed with primary or secondary progressive multiple sclerosis (PPMS or SPMS, two men and six women) were included in the qualitative immunohistochemical analyses. Clinical and demographic characteristics are summarized in Table 1.

**Table 1:** Clinical and demographic data of progressive multiple sclerosis donors.

Donor	Clinical diagnosis	Age	Sex	PMD (hours)	pH of CSF	Brain weight (grams)	Disease duration (years)	Cause of death	Tissue	Fixation
**MS-1**	PPMS	50	F	07:45	6.7	1096	17	Legal euthanasia	CTX lesion	FFPE
**MS-2**	PPMS	49	F	08:30	6.3	1080	25	NA	CTX lesion	FFPE
**MS-3**	SPMS	48	F	09:20	6.1	1175	24	NA	CTX lesion	Cryo
**MS-4**	SPMS	70	M	09:25	7.2	1390	38	Legal euthanasia	CTX lesion	Cryo
**MS-5**	PPMS; ON	56	M	09:35	6.4	1245	33	End stage MS with urosepsis	Optic nerve	FFPE
**MS-6**	SPMS; ON	54	F	07:00	6.7	1242	37	Pneumonia/ urinary tract infection	Optic nerve	Cryo
**MS-7**	SPMS; ON	57	F	10:40	6.8	1145	28	Legal euthanasia	Optic nerve	Cryo
**MS-8**	SPMS; ON	53	F	07:16	6.5	998	18	Pneumonia	Optic chiasm	Cryo

Abbreviations used in Table 1: Cryo, cryotissue; CTX, neocortex; F, female; FFPE, formalin-fixed paraffin-embedded; M, male; PMD, postmortem delay; CSF, cerebrospinal fluid; MS, multiple sclerosis; ON, optic neuritis; PPMS, primary progressive multiple sclerosis; SPMS, secondary progressive multiple sclerosis.

#### Classification of demyelinating lesions.

Immunohistochemical analysis was employed to identify key histopathological features necessary for the classification of MS lesions. Double immunostaining was performed on sections from all dissected tissue blocks to visualize proteolipid protein (PLP) and human leukocyte antigen (HLA-DR-DQ, hereafter referred to as HLA) as previously described by the Neuroimmunology Research Group at the Netherlands Institute for Neuroscience [50,51]. White matter and cortical grey matter MS lesions were characterized according to the system established by Kuhlmann et al. [52]. Innate inflammatory lesion activity was determined based on the presence of HLA+ microglia/macrophages. White matter lesions were discriminated against normal white matter based on demyelination and the presence of HLA+ microglia/macrophages, while cortical grey matter lesions, identified as demyelinated areas, were classified by location. The criteria for both lesion types were used as described [51].

#### Histology on human postmortem tissue samples.

For cryosectioning, coronal sections (8 µm thick) were cut using a cryostat. After applying a liquid blocker, the sections were fixed in 4% paraformaldehyde in phosphate-buffered saline (1x PBS, pH 7.6) and incubated in a permeabilization solution (1% H_2_O_2_, 0.2% Triton X-100 in 1x PBS). After blocking with a solution containing 10% normal horse serum (NHS), 1% bovine serum albumin (BSA), and 0.5% Triton X-100 in 1x PBS, the sections were incubated overnight at 4°C in blocking solution containing the primary antibodies.

For formalin-fixed paraffin-embedded (FFPE) tissue, sections were first deparaffinized and processed for antigen retrieval in either 10 mM Citrate buffer pH 6.0 (for GPR17) or 1x TBS pH 7.6 (for HLA and PLP). Next, endogenous peroxidase activity was quenched with a solution of 1% H_2_O_2_ and 0.2% Triton X-100 in 1x TBS, followed by blocking and incubation of primary antibodies as described for cryosections.

The primary antibodies used were the following: Mouse anti-human HLA-DP, DQ, DR antigen (DAKO, M077501-2; FFPE: 1:100, Cryo: 1:500); mouse anti-PLP (Bio-Rad, MCA839G; FFPE: 1:500, Cryo: 1:6000), rabbit anti-GPR17 (Sigma-Aldrich, HPA026; FFPE: 1:100, Cryo: 1:100), mouse anti-BCAS1, clone 5 (Santa Cruz, sc-136342; FFPE and Cryo 1:10’000).

The following day, for the HLA and GPR17 stainings, the sections were incubated in biotinylated secondary antibodies (horse anti-mouse or anti-rabbit, Vector Laboratories) at 1:400 in blocking solution, while for PLP staining, HRP-mouse EnVision kit (DAKO, K5007) was used. For all antibodies, sections were incubated in DAB EnVision kit (1:50), rinsed in tap water, and dehydrated through an ethanol and xylene series, and mounted with Entellan (Sigma-Aldrich).

Sections depicted for double immunofluorescence stainings were incubated in the following secondary antibodies diluted in blocking solution: Donkey anti-mouse IgG Cy3 at 1:600, and donkey anti-rabbit IgG DyLight 649 at 1:800. After secondary antibody incubation, sections were incubated in Hoechst (1:1000 in 1x PBS pH 7.6), followed by 0.1% Sudan Black and by mounting in Mowiol (EMD Chemicals).

#### Image acquisition and qualitative assessments.

Brightfield images of 3,3-diaminobenzidine (DAB)-stained sections were acquired using an Axio Scan Z.1 microscope (Zeiss) equipped with Neoplanfluor objectives to capture entire MS lesions. Images were recorded with an Evolution MP camera (Media Cybernetics) and processed using ImagePro software. Fluorescent images were obtained using an Axio Scan Z.1 (Zeiss) with Plan-Apochromat 20x/0.8 M27 objective. An Evolution black-and-white camera (MediaCybernetics) and ImagePro software (MediaCybernetics) were used for image capture and analysis, respectively. For each donor and tissue block dissected (neocortex and optic nerves), all visibly distinct lesions were included in the qualitative assessment. A total of 3–4 sections per block were included and all lesions (2–6 per section) that were visibly distinct were evaluated separately.

### RWT020016 synthesis and pharmacokinetics

The test compound RWT020016 was synthesized by the CRO Pharmaron Inc. via standard solution‑phase synthesis from commercially available building blocks using established coupling, substitution, and protection–deprotection strategies, followed by chromatographic purification and routine analytical characterization. The GPR17 antagonists belong to a class of pyrrolyl- and fused pyrrolyl-sulfonamide derivatives synthesized via modular routes that enable systematic diversification of the heterocyclic core and appended aryl/heteroaryl moieties. These compounds were obtained as pharmaceutically relevant forms (e.g., salts, solvates, polymorphs) and were designed to balance lipophilicity and polarity to support CNS exposure. Structural optimization further enabled favourable pharmacokinetic properties, including high oral bioavailability and good brain penetration. Full synthetic procedures are described in patents WO2022254027 (3‑Pyrrolylsulfonamide compounds as GPR17 antagonists, published December 8, 2022) and WO2024115733 (Fused pyrrolyl‑sulfonamide compounds for the treatment of GPR17‑mediated disorders, published June 6, 2024) [53,54]. At this stage of development, the chemical structure of the test compound RWT020016 cannot be disclosed by Rewind Therapeutics NV owing to strategic and legal constraints.

Single-dose pharmacokinetic studies (1 day experiment) as well as brain exposure analyses were performed by the CRO Pharmaron Inc. using standard industry NeuroPK protocols (N = 3, dose volume of 5 ml/kg). Plasma samples were obtained by terminal cardiac puncture, while brain tissues were collected following exsanguination and saline perfusion to minimize residual blood contamination. Plasma and brain homogenate concentrations were quantified using a validated LC-MS/MS method. Pharmacokinetic parameters, including bioavailability (%F), maximum concentration (C_max,brain_), time to maximum concentration (T_max,brain_), terminal half-life (*t*_1/2_), area under the concentration–time curve (AUC_**0-24h,brain**_), and brain-to-plasma ratios were calculated using noncompartmental analysis.

### *In vitro* potency and selectivity assays

#### cAMP Homogeneous Time-Resolved Fluorescence (HTRF) potency assay.

cAMP accumulation assays were performed by the CRO Eurofins DiscoverX (Celle-Lévescault, France) using CHO-K1 cells stably expressing the recombinant murine GPR17 receptor (Eurofins-DiscoverX_GPCRs-TargetList.pdf). Dose-response curves for RWT020016 were generated at eight concentrations, each tested in duplicate. For antagonist testing, cells were preincubated with RWT020016 for 10 min, followed by the addition of a mixture containing forskolin (1 µM) and the GPR17 agonist MDL-29951 (EC₈₀, 200 nM). After 30 min incubation at room temperature, lysis buffer containing cAMP-d_2_ and anti-cAMP cryptate detection reagent (Cisbio #62AM4PEJ) was added. Plates were incubated for 1 h at room temperature, and fluorescence resonance energy transfer (FRET) ratios were measured using the HTRF kit, according to the manufacturer’s protocol.

#### Calcium mobilization-based assays.

Calcium mobilization–based potency and selectivity assays were performed by the CRO Axxam S.p.A. (Bresso, Italy) using the Fluorescence Imaging Plate Reader FLIPR TETRA ICCD camera (Molecular Devices) including the selective GPR17 agonist, MDL-29951. Additional automated liquid handling systems included the Hamilton Microlab STAR (Hamilton) and CyBio Felix and CyBio Well Vario systems (CyBio).

For potency assessment, HEK293 cells stably expressing either the murine or human short GPR17 isoform were loaded with 20 µL per well of 0.5 × calcium-sensitive dye (Fluo-8 NW; AAT Bioquest) in Tyrode’s buffer and incubated for 1 h at 37 °C. Cells were treated with RWT020016 in a dose–response format for 20 min, then stimulated with MDL-29951 (EC₈₀, 200 nM). Vehicle-treated cells served as controls.

For selectivity testing, human P2Y_1_, P2Y_2_, and P2Y_1__1_ receptors were transiently expressed in 1321N1 cells, and human CysLTR_1_ was transiently expressed in HEK293 cells. The corresponding wild-type cell lines served as negative controls. Cells were loaded with Fluo-8 NW dye as described above, treated with RWT020016 in a dose-response format for 20 min, and stimulated with reference agonists at EC₈₀ concentrations (ATP for P2Y receptors, LTD_4_ for CysLTR_1_). Additional control conditions included treatment with reference antagonists, MRS2500 (P2Y_1_), ARC-118925XX (P2Y_2_), Suramin (P2Y_1__1_), and Pranlukast (CysLTR_1_), in a dose–response format, followed by stimulation with the corresponding reference agonists. Fluorescence signals were recorded in real time, and data were analyzed using Genedata Screener® software (Genedata AG) and expressed as a percentage effect relative to normalization standards.

### Animals

Cuprizone experiments were conducted at the Hasselt University animal facility under a facility access agreement with Rewind Therapeutics and in accordance with protocols approved by the local Ethical Committee (matrix ID: 202137) and EU Directive 2010/63/EU. Nine-week-old male C57BL/6OlaHsd mice (Envigo/Inotiv) were single housed under standard enriched conditions with ad libitum access to food and water, controlled temperature (21–22°C),and an inverted light cycle. Animal health and behavior were monitored daily by trained personnel.

NeuroPK experiments were conducted at the CRO Pharmaron Inc. using SPF-grade male C57/BL6J mice (Zhejiang Vital River Laboratory Animal Technology Co., Ltd, license number: SCXK (浙) 2020–0002). Animals were maintained under a reverse 12-h light/dark cycle with ad libitum access to food and water. All procedures were approved by the Pharmaron Institutional Animal Care and Use Committee (IACUC). At study end, animals were euthanized with pentobarbital (200 mg/kg i.p.).

### Cuprizone-induced demyelination models and compound treatment

Following a one-week acclimatization period, mice were randomly assigned to either a control or cuprizone-treated group. Cuprizone-induced demyelination was achieved by feeding mice a pelleted diet containing 0.3% (w/w) cuprizone (Bis(cyclohexanone)oxaldihydrazone; Sigma-Aldrich, C9012-25G), formulated by Bio-Services/Sniff. Control animals received an identical standard diet without cuprizone. Cuprizone treatment was administered for either 6 weeks (acute model) or 12 weeks (chronic model), based on established protocols [55,56].

Body weight was monitored five times per week during the cuprizone treatment phase and daily from the initiation of compound or vehicle administration. Animals exhibited a modest cuprizone‑related weight loss of, on average, 9–15% during the first few days of treatment. Following this initial decrease, all animals either maintained a stable body weight or showed some gradual weight gain for the remainder of the study. Importantly, no animal’s body weight fell below 20% of its baseline value, remaining well above the humane‑endpoint threshold of 30% weight loss approved by the ethics committee, which would have required immediate euthanasia via pentobarbital injection (200 mg/kg, i.p.). No animals needed to be removed from the study for health‑related reasons, nor did any animals die in either the acute or chronic cuprizone studies. Compound or vehicle treatment began 2 days before cessation of cuprizone feeding in both models. This timing was chosen to ensure sufficient brain exposure of the compound at the onset of the remyelination phase, when endogenous repair processes begin to dominate. Importantly, demyelination in the cuprizone model is not a strictly discrete process but rather reflects a dynamic overlap of ongoing demyelination and early remyelination responses that may already be initiated while the toxin is still present. This dosing paradigm was based on previous pilot studies indicating robust treatment effects under these conditions.

Mice were treated once daily via oral gavage (10 µl/g body weight) for 9 days (acute model) or 37 days (chronic model). The selective GPR17 antagonist RWT020016 was dissolved in vehicle, consisting of 1% (v/v) DMSO, 0.5% (w/v) methylcellulose (M0512, Sigma-Aldrich) and 2% (v/v) Tween 80 (P1754, Sigma-Aldrich) and delivered at doses of 0.3 mg/kg and 1 mg/kg.

### Spontaneous Y-maze alternation task

Spatial working memory was assessed using the spontaneous Y-maze alternation task at the end of the demyelination (week 6 and week 12) and the remyelination (week 7 and week 17) period in the acute and chronic cuprizone model, respectively, as previously published [57]. In brief, each mouse was placed in one of the arms of the Y-maze facing the center and allowed to freely explore all three arms for a total of 6 minutes. An arm entry was recorded by the experimenter, who was blinded to the condition, when both hind paws of the mouse fully entered an arm. A correct triad was defined as a sequence of consecutive entries into three different arms (e.g., ABC, CAB, BCA). The primary measure of spatial working memory was the percentage of spontaneous alternation, calculated using the formula:


$$\begin{array}{c} \left(\frac{Number\;of\;correct\;triads}{total\;arm\;entries-\text{2}}\right)x\text{1}\text{0}\text{0} \end{array}$$

Spontaneous alternation in the Y-maze is commonly used as a measure of short-term spatial working memory, with chance performance corresponding to approximately 50% alternation under the standard triad-based scoring approach, consistent with recent behavioural studies [57–60]. Therefore, alternation percentages significantly above 50% were interpreted as evidence of intact working memory. Data were analyzed by an experimenter blinded to treatment groups for both the initial 2-minute trial for the acute cuprizone model and the entire 6-minute trial for the chronic cuprizone model. Mice that failed to complete at least five triads during the 6-minute trial were excluded from analysis due to insufficient exploration.

### Visual evoked potential (VEP) recordings

To assess visual pathway function, mice underwent non-invasive VEP recordings at baseline, at week 6 (end of cuprizone treatment) and week 7 (after 7 days of compound/vehicle treatment) in the acute model, and at week 12 (end of cuprizone treatment) and week 17 (after 35 days of compound/vehicle treatment) in the chronic model. Prior to testing, animals were dark-adapted overnight to enhance retinal light sensitivity. Anesthesia was induced via intraperitoneal injection (5 µl/g body weight) of xylazine (10 mg/kg, Rompun), ketamine (70 mg/kg, Anesketin, Eurovet), dissolved in saline. Pupillary dilation was achieved using 0.5% tropicamide (Monofree Tropimide, Thea Pharma), followed by 10% phenylephrine (Thea Pharma). For electrophysiological setup, a subdermal recording electrode was inserted over the visual cortex, with a reference electrode placed in the tongue and a ground electrode at the base of the tail to minimize electrical interference. Saline was applied to maintain corneal hydration, and flash electrodes were placed on the eyes. Electrode connectivity was confirmed using impedance measurements prior to data acquisition. Each mouse was exposed to 200 white light flashes (310 ms duration, 1 Hz frequency) at an intensity of 0.5 cd·s/m². N1 VEP latency (measured in milliseconds) was defined as the time interval between stimulus onset and cortical response, serving as an indirect readout of visual pathway myelination, while N1-P1 amplitude (measured in microvolts) was assessed to investigate potential axonal damage. All recordings were performed in complete darkness inside a Faraday cage using the CELERIS system (Diagnosys). After electrode removal, eye ointment (Vidisic) was applied to prevent corneal drying and avoid any distress. Data were analyzed using Diagnosys ESPION software (Diagnosys). For each eye, VEP traces were analyzed offline by identifying the N1 peak latency and measuring the N1–P1 peak-to-peak amplitude. Data were analyzed by an experimenter blinded to treatment groups. Values from both eyes of each mouse were averaged and used for statistical analysis.

### Immunohistochemistry on brain sections

Mice were perfused intracardially with saline after a 200 mg/kg pentobarbital (i.p.) injection, and brains were isolated and post-fixed overnight in 4% PFA. After incubation in a graded sucrose series (10%, 20% and 30%), the brains were embedded in optimal cutting temperature medium (Tissue Tec, VWR), and coronal cryosections (5 µm) were prepared for both acute and chronic brains, with coronal sections selected at Bregma −2.0 mm. Cryosections were fixed in ice-cold acetone and sections were then incubated with blocking buffer (0.05% Triton X-100, Thermofisher Scientific + 10% DAKO protein block in PBS, DAKO), followed by overnight incubation with the primary antibodies rat anti-myelin basic protein (MBP; MAB386, Millipore, 1:500) or rabbit anti-GPR17 (10136, Sanbio Cayman Chemicals, 1:200), both diluted in blocking buffer and afterwards incubated with anti-rat Alexa 555 (A21434, Life Technologies, 1:400) or anti-rabbit Alexa 555 (A31572, Life Technologies, 1:400) secondary antibodies respectively for MBP or GPR17, both diluted in 1x PBS. DAPI was used to counterstain the cell nuclei and slides were mounted using Fluoromount G (00-4958-02, Life Technologies). Representative high-resolution images of both MBP- and GPR17-stained sections were made with a Leica DM2000 LED fluorescence microscope. A densitometric method was used to quantify the MBP and GPR17 immunoreactivity. All analyses were performed blinded in the medial corpus callosum (CC) at Bregma level −2.0 mm. For each image, a 32-image format was used to apply a binary threshold with the default ImageJ (Fiji) thresholding plugin, separating the immunoreactive signal from background. The area above threshold (area fraction) within the region of interest (a square of fixed dimensions) was measured and used to calculate the % area stained. For each mouse, an average value of the MBP-/GPR17-positive stained area was obtained. Values from the cuprizone and treatment groups were expressed relative to the control group.

### Transmission electron microscopy on optic nerve sections and quantification

Following a lethal injection with 200 mg/kg pentobarbital (i.p.) and subsequent cardiac perfusion, mouse optic nerves were dissected and fixed at week 17 of the chronic cuprizone study, as previously described [61]. In brief, the optic nerves were immersed overnight at 4°C in fixative (2% glutaraldehyde in 0.05 M sodium cacodylate buffer, pH 7.3). Next, the optic nerves were washed in 0.1 M sodium cacodylate buffer and post-fixed in 2% osmium tetroxide in 0.1 M sodium cacodylate buffer for 60 minutes. After dehydration through ascending concentrations of ethanol, the optic nerves were incubated in propylene oxide for 60 minutes, followed by overnight infiltration with a 1:1 mixture of propylene oxide and epoxy resin. The following day, the optic nerves were embedded in Epon and polymerized at 60°C for 48 hours. Ultrathin cross-sections (60–80 nm) were cut on a Reichert Ultracut microtome, mounted on copper grids and stained with lead citrate. Images were acquired using transmission electron microscopy (TEM). For each optic nerve, five images of optic nerve sections per mouse were randomly selected and in a blinded manner analyzed using Fiji ImageJ software. The g‑ratio was calculated as:


$$\begin{array}{c} g-ratio=\frac{d\text{ axon}}{d\;fiber} \end{array}$$

where *d*_*axon*_ is the inner axonal diameter and *d*_*fiber*_ is the total outer diameter of the myelinated fiber (including the myelin sheath). G‑ratios were measured for 25 axons per image to assess myelin thickness using Fiji (ImageJ). Ultrastructural pathology was assessed using a semi-quantitative TEM-based scoring system (Table 2). In a previous pilot study, the numerical density of axons containing mitochondria was assessed within 5 images of 200 μm^2^ size per animal (n = 5). It yielded an average of 0.15/μm^2^ (equivalent to 30 per 200 μm^2^). This value was used as a reference for normal mitochondrial density and morphology in the current study.

**Table 2:** Scoring schematic to grade pathology of the optic nerve.

Score	Characteristics
**0**	No evidence for altered mitochondria, No loose myelin sheaths, No empty/swollen axons, No existing degraded axons with only myelin sheaths left, No degenerating axons with cellular/membranous debris in axon
**+1**	Appearance of mitochondria with clearly altered morphology
**+1**	Higher density of mitochondria with altered morphology compared to reference value
**+1**	Loose myelin sheath
**+1**	Empty/swollen axon(s)
**+1**	Degenerating axons with cellular/membranous debris in axon
**+1**	Degenerated axons with only myelin debris left
**+1**	Enlarged periaxonal space

For each image, a baseline score of 0 was assigned when no pathological features were present, defined as the absence of altered mitochondrial morphology, loose myelin sheaths, empty or swollen axons, enlarged periaxonal spaces, or evidence of axonal degeneration. A cumulative pathology score was generated by adding +1 for the presence of each of the following features: (i) appearance of mitochondria with altered morphology and higher density of mitochondria with altered morphology compared to reference value, (ii) loose myelin sheaths, (iii) empty or swollen axons, (iv) degenerating axons containing cellular or membranous debris, (v) fully degenerated axons with only myelin debris remaining, and (vi) enlarged periaxonal space. This approach allowed multiple pathological hallmarks to be captured within a single image, providing an integrated measure of ultrastructural damage. The average score per optic nerve was used to classify samples into grade 1 (score 2–3, none), grade 2 (score 3–4, mild), grade 3 (score 4–5, moderate), and grade 4 (score 5–6, severe).

### Statistical analysis

All analyses were performed using GraphPad Prism for Windows (version 10.2.1). The specific statistical tests applied to each dataset are reported in the corresponding figure legends. Data are presented as mean values ± standard error of the mean (SEM). Differences were considered statistically significant at *p* < 0.05.

## Results

### GPR17 expression in committed oligodendrocyte precursors is conserved in human brain

**Fig 1:**
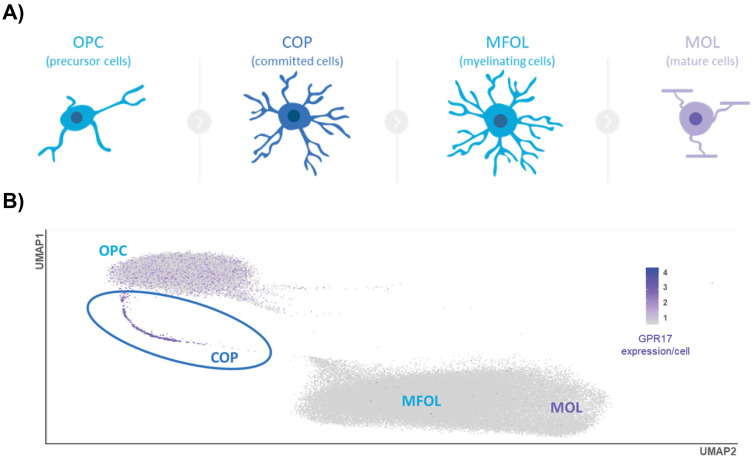
GPR17 is highly expressed in committed oligodendrocyte precursor cells. (A) Schematic representation of the oligodendrocyte lineage progression from oligodendrocyte precursor cells (OPCs), through COPs and myelinating oligodendrocytes (MFOLs), to fully mature oligodendrocytes (MOLs). (B) UMAP (Uniform Manifold Approximation and Projection) plot visualizing single nucleus RNA sequencing data of the oligodendrocyte lineage, which represents a combined dataset from five different human brain studies. Cells are embedded according to transcriptional similarity along UMAP1 and UMAP2 axes. GPR17 expression per cell is shown as a color gradient (purple), with highest expression localized specifically to the COP population. OPCs show low expression, while more differentiated MFOLs and MOLs show minimal or no GPR17 expression.

Single-nucleus transcriptomic analysis of oligodendrocyte lineage cells, combined from five human brain studies, was performed with a special emphasis on markers of oligodendrocyte maturation [5,43–46]. A total of 214,567 cells were included in the analysis, with a population of 71,623 cells of the oligodendrocyte lineage. Uniform Manifold Approximation and Projection (UMAP) plots were used to visualize major CNS cell populations (S1A-B Fig), including astrocytes, microglia, neurons, oligodendrocytes and OPCs. Cluster identities were validated using canonical marker genes for microglia (FYB1, C3, S1C-D Fig), astrocytes (AQP4, GJA1, S1E-F Fig), oligodendrocytes (MOBP, MBP, S1G-H Fig), neurons (GRIN1, GAD2, SLC17A7, S1I-K Fig), and endothelial cells (CLDN5, S1L Fig). Lineage progression from OPCs to NFOLs was confirmed by the expression of stage-specific markers (SOX6, BMP4, TNS3, TCF7L2, CASR, FYN, ITPR2, S1O-V Fig). The analysis revealed that GPR17 expression was largely restricted to the oligodendrocyte lineage, with no detectable transcripts in other major CNS cell types, including astrocytes, microglia, neurons, or endothelial cells (S1 Fig). Sample-based integration of non-neuronal cells minimized dataset- and sample-specific variation, enabling clear delineation of the rare and transient population of committed oligodendrocyte precursors (COPs). These COPs were characterized by relatively higher GPR17 expression compared to OPCs, alongside the presence of additional markers associated with early differentiation stages, including FYN kinase and myelin-related genes such as PLP1 and MBP [62]. Other COP-associated markers (e.g., SOX6, TNS3, FYN) showed enrichment but were not exclusively restricted to this population (S1O, R, U Fig). As illustrated in the UMAP projection (Fig 1B), GPR17 expression appeared to peak within the COP population and decline as cells transitioned toward myelin-forming and mature oligodendrocytes (S1Q Fig). While these observations are primarily descriptive, they consistently support an enrichment of GPR17 expression at the COP stage relative to adjacent maturation states. A smaller subset of OPCs also exhibited detectable GPR17 expression, albeit at lower levels. Together, these findings highlight a transient yet prominent association of GPR17 expression with the COP maturation stage in the human brain.

### GPR17 is upregulated in pre-myelinated BCAS1 + COPs in vicinity of demyelinating lesions

**Fig 2:**
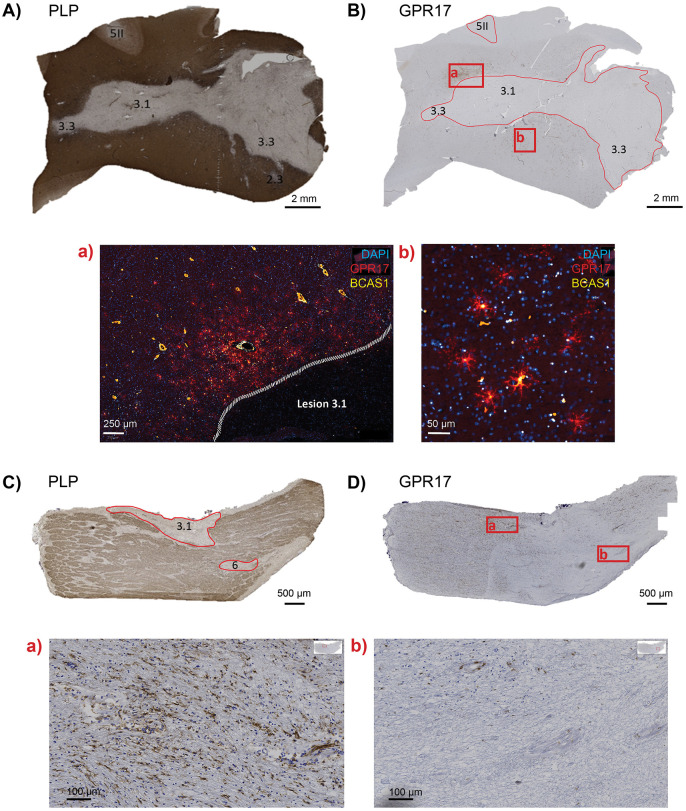
GPR17 expression in oligodendrocyte lineage cells is selectively associated with demyelinated MS lesions. (A-B) Representative images of brain sections stained for proteolipid protein (PLP) (A) to visualize myelin and for GPR17 (B) to localize GPR17-expressing cells. Lesions were classified according to a standard MS lesion scoring system (codes 0–6): Lesion 3.1 is a mixed active/inactive lesion with ramified microglia/macrophage morphology (code 3), surrounded by normal-appearing white matter (NAWM, code 0), additional mixed active/inactive lesions (lesion 3.3, classified as foamy microglia/macrophage morphology), and a cortical grey matter lesion (code 5II). (a,b) High-resolution images of GPR17 co-staining with BCAS1 and DAPI in lesion 3.1. GPR17 expression is observed in BCAS1^+^ COPs at the lesion edge (area a), and outside the lesion core (area b). (C,D) Optic nerve sections stained for PLP (C) and GPR17 (D), highlighting lesion 3.1 (code 3) and a shadow plaque (lesion 6). (a,b) High-magnification views of boxed areas in (D): area a (within demyelinated lesion) shows abundant GPR17^+^ cells, while area b (shadow plaque/remyelinated lesion) shows minimal to no GPR17 expression. Scale bars are depicted in the figure panels.

To qualitatively investigate the spatial relationship between GPR17 expression and MS lesion pathology, we investigated brain and optic nerve sections stained for GPR17, HLA/PLP, and BCAS1 (Brain Enriched Myelin Associated Protein 1, a marker of pre-myelinating oligodendrocytes). Lesions were classified according to established histopathological criteria published by Luchetti et al. [51] based on demyelination status and microglia/macrophage activity. In brain tissue (Fig 2 A-B), HLA/PLP staining delineated multiple lesion types. Lesion 3.1, classified as a mixed active/inactive lesion characterized by microglia/macrophages with a ramified morphology, showed reduced myelin content and a characteristic hypocellular core surrounded by an active microglial rim. Within and adjacent to these demyelinating lesions, GPR17^+^ cells were readily observed (Fig 2 B). High-magnification imaging (Fig 2 B′) revealed that a subset of these GPR17^+^ cells co-expressed BCAS1, indicating they were COPs in a pre-myelinating state. These GPR17^+^/BCAS1^+^ cells were particularly enriched near the lesion borders. In contrast, GPR17 expression was minimal or not detectable in areas that appeared fully remyelinated, suggesting a spatial association of GPR17-expressing COPs with active or chronic demyelinated lesions, but not with successfully remyelinated regions. Analysis of the optic nerve (Fig 2 C-D), including lesion 3.1 (code 3) and lesion 6 (shadow plaque), further supported this pattern observed in the brain. Indeed, GPR17 immunoreactivity was predominantly observed in association with demyelinated zones (Fig 2 D), particularly localized at the lesion edges. High-magnification imaging (Fig 2 D′) illustrated the presence of GPR17-expressing cells within demyelinated areas (area 1), whereas GPR17 immunoreactivity was not detected in regions classified as shadow plaques (area 2), indicative of partial remyelination (area 2) [63]. This finding supports similar observations made in cortical regions. Of note, due to limited sample numbers and inter-patient heterogeneity, no formal quantitative comparisons across lesion types were performed as this type of quantitative comparisons across lesion types would not yield statistically robust or biologically meaningful results. Together, these descriptive observations suggest that GPR17-expressing COPs persist in association with demyelinated lesions, even in chronic disease stages such as secondary progressive MS, supporting their potential relevance as pharmacological targets for remyelination.

### Cuprizone-induced demyelination reveals strong GPR17 upregulation in the corpus callosum

**Fig 3:**
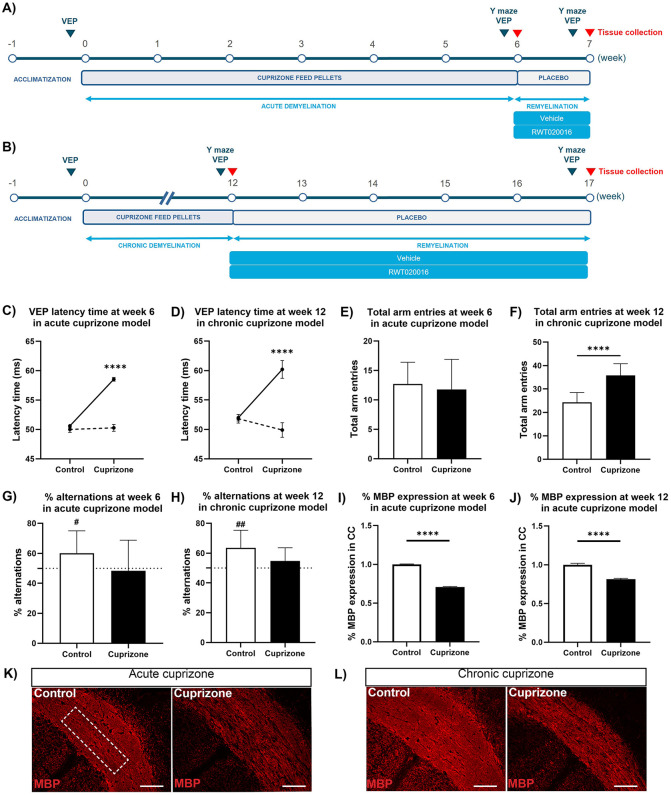
The acute and chronic mouse cuprizone model as mechanistic models to study demyelination. (A, B) Experimental timelines for the acute (A) and chronic (B) mouse cuprizone model. (C,D) Functional demyelination was evaluated by visual evoked potential (VEP) latency at the end of the demyelination phase, showing a significant delay in latency time at week 6 (p < 0.0001 vs. control, Two-way ANOVA with Dunnett’s multiple comparisons test, N = 10) in the acute (C) and at week 12 (p < 0.0001 vs. control, Two-way ANOVA with Dunnett’s multiple comparisons test, N = 5-10) in the chronic model (D). (E, F) Similarly, total number of entries in the spontaneous alternation Y-maze test was performed at the end demyelination phase, showing no difference in the acute model (N = 11), while chronic cuprizone exposure resulted in increased locomotor activity (p < 0.0001 vs. control, unpaired t-test, N = 10-11). (G, H) Percentage of spontaneous alternations in the Y-maze at week 6 (G) and week (H). Both acute and chronic cuprizone exposure resulted in reduction in alternation performance, indicating impaired spatial working memory (*p* = 0.04 for acute, *p* = 0.0003 for chronic, one-sample t-test vs. hypothetical 50% alternation, N = 10-11). (I,J) Quantifying MBP-positive area in the medial corpus callosum (CC) following acute (I) and chronic (J) cuprizone demyelination showed significantly decreased MBP expression (p < 0.0001 vs. control for both models, unpaired t-test, N = 5-10). (K,L) Representative MBP immunofluorescence images of the medial CC in control and cuprizone-treated mice in the acute (K) and chronic (L) model. The dotted-line square in the acute control panel indicates the region of medial CC used for quantitative analysis. Data are presented as mean ± SEM. #*p* < 0.05, ##*p* < 0.01, ****p < 0.0001. Scale bars: 100 µm (K,L).

The effect of GPR17 antagonism on remyelination was further examined using the acute (0.3% for 6 weeks) and chronic (0.3% for 12 weeks) cuprizone model. After cuprizone administration, animals were returned to normal placebo pellets to allow spontaneous remyelination for 1 week and 5 weeks respectively. Compound treatment was initiated two days prior to cuprizone withdrawal to ensure sufficient compound concentration in the brain to initiate remyelination (Fig 3 A-B). To evaluate functional recovery and remyelination following cuprizone-induced demyelination, we assessed visual evoked potential (VEP) latency and amplitude in both the acute and the chronic model. In clinical practice, this method is used to assess the functional integrity of the visual pathways, particularly in the context of demyelinating disease, such as MS. Demyelination slows the conduction of action potentials, which is reflected as a prolongation of VEP latency, while a reduction in amplitude may indicate axonal injury or sustained demyelination [64]. VEPs were assessed at baseline, at the end of the demyelination phase (at week 6 for acute and at week 12 for chronic) and at the end of the remyelination phase (at week 7 for acute and at week 17 for chronic). At the end of the demyelination phase, both models showed a significant increase in VEP latency (*p* < 0.0001 vs. control for acute; *p* < 0.0001 vs. control for chronic, Fig 3 C-D), while VEP amplitudes remained comparable to those of control mice, indicating the absence of axonal loss in either model (data not shown), in line with previous findings [65].

In addition, the impact of demyelination on axonal signal conduction can manifest in deficits in cognitive performance. Cognitive processing speed and episodic memory, including short-term working memory, are the most frequently affected cognitive domains in patients with MS [66]. Therefore, mice were subjected to the Y-maze spontaneous alternation test at the end of the demyelination phase. To rule out confounding effects on cognitive performance, the effects of cuprizone on general locomotor activity were also investigated. At week 6, no significant difference was observed in the total arm entries between control and cuprizone-treated mice (Fig 3 E) indicating that cuprizone did not affect general locomotor activity. However, at week 12, cuprizone-treated mice showed a significant increase in total arm entries compared with controls (*p* < 0.0001 vs. control, Fig 3 F), indicating hyperactivity or altered exploratory behavior following chronic cuprizone treatment. Furthermore, in both models, cuprizone-treated mice showed a reduction in spontaneous alternation compared with control mice, indicative of impaired working memory due to demyelination (Fig 3 G-H). Furthermore, to investigate structural changes in myelin following acute and chronic cuprizone exposure, MBP immunohistochemical expression was evaluated in the medial CC (Fig 3 K-L). Quantitative analysis of MBP expression revealed a significant reduction in MBP-positive staining after the demyelination phase in both the acute (*p* < 0.0001 vs. control) and chronic (*p* < 0.0001 vs. control) model, confirming the extensive loss of myelin induced by cuprizone (Fig 3 IJ).

**Fig 4:**
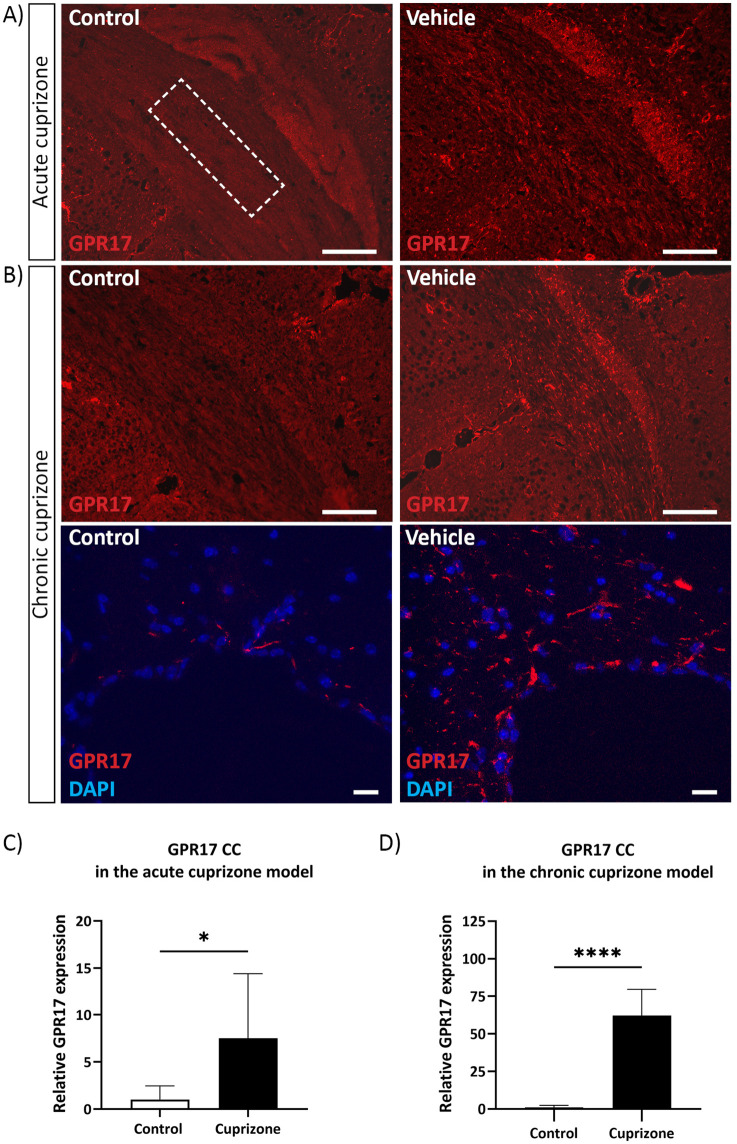
GPR17 expression is upregulated in the corpus callosum following cuprizone-induced demyelination. (A) Representative images of coronal brain sections showing GPR17 immunoreactivity (red) in the CC of control and vehicle-treated mice following 6 weeks of cuprizone treatment (acute model). The dotted-line square in the acute control panel indicates the region of medial CC used for quantitative analysis. (B) Top panel: Images of coronal sections showing GPR17 protein expression in the chronic cuprizone model, following 12 weeks of demyelination. Bottom panels: Higher-magnification images of sagittal sections showing GPR17 (red) and DAPI (blue) staining in the CC of control and cuprizone-treated mice following chronic demyelination. (C) Quantification of relative GPR17 protein expression in the CC of control and cuprizone-treated mice in the acute cuprizone model, showing significant upregulation of GPR17 expression (*p* = 0.01 vs. control, unpaired t-test, N = 9-11). (D) Quantification of relative GPR17 protein expression in the chronic cuprizone model revealed significant upregulation following cuprizone treatment (*p* < 0.0001 vs. control, unpaired t-test, N = 9-11). Data are presented as mean ± SEM and are fold-change relative to control mean (mean control values are set to 1.0); *p < 0.05, ****p < 0.0001. Scale bars: 100 µm (A,B).

To determine whether these functional and structural changes were associated with altered GPR17 expression in areas undergoing demyelination, we performed immunohistochemical analysis of GPR17 expression in the medial CC. As shown in Fig 4 A, GPR17 expression was markedly increased in the CC following acute and chronic cuprizone exposure compared with control. Quantitative morphometric analysis confirmed a significant upregulation of GPR17 expression in both cuprizone models (*p* = 0.01 vs. control for acute; *p* < 0.0001 vs. control for chronic) (Fig 4 B-C).

### RWT020016 is a potent, selective and brain penetrant GPR17 antagonist

RWT020016 is an undisclosed small molecule selected in the lead optimization of a series of sulfonamides recently published by Rewind Therapeutics [53,54].

To evaluate the inhibition of downstream signaling of GPR17, we assessed the potency of RWT020016 in cAMP accumulation assays across species and GPR17 isoforms in the presence of the synthetic agonist, MDL-29951. As summarized in Table 3, RWT020016 demonstrated potent activity on human GPR17, with an IC_50_ of 21 nM for the short isoform (s-GPR17) and 34 nM for the long isoform (l-GPR17), indicating no substantial difference in potency between the two variants. For murine GPR17, RWT020016 showed reduced potency compared with the human isoforms with an IC_50_ of 225 nM. Similar potencies of RWT020016 were measured in the calcium mobilization assay, with IC_50_ values between 60 and 75 nM for s-GPR17 Gαi and Gαq signaling pathways, respectively. In this assay as well, potency on the murine GPR17 receptor was reduced (142 nM) compared with the human receptor. While this represents approximately a 2- to 10-fold decrease in potency across assays and species, the compound still falls within the submicromolar range and is therefore considered a potent GPR17 antagonist, which supports its continued use in our preclinical cuprizone models. As indicated in Table 3, RWT020016 was only very weakly active on mock-transfected cells in the calcium mobilization assay, confirming that its observed activity is specific to GPR17.

**Table 3:** Potency of RWT020016 across human and mouse GPR17 receptors.

Assay	GPR17 isoforms	IC_50_
**Calcium** **mobilization assay**	Human s-GPR17 G_αi_ Wild Type G_αi_	60 nM 12.6 μM
	Human s-GPR17 G_αq_ Wild Type G_αq_	75 nM >12.5 μM
	Mice GPR17 G_αq_	142 nM
**cAMP assay**	Human s-GPR17	21 nM
	Human l-GPR17	34 nM
	Mice GPR17	225 nM

Abbreviations used in Table 3: s-GPR17, short-GPR17 isoform; l-GPR17, long-GPR17 isoform.

In addition, the selectivity profile of RWT020016 was evaluated against the closest related Class A GPCRs – including cysteinyl leukotriene receptors (hCYSLTR1) and multiple purinergic P2Y family members (P2Y_1_, P2Y_2_, P2Y_11_). RWT020016 showed no measurable activity at these receptors at concentrations up to 50 µM, demonstrating high selectivity for GPR17 (Table 4).

**Table 4:** Selectivity of RWT020016 against closely related Class A GPCRs in calcium mobilization assays.

Selectivity assays	IC_50_
Human cysteinyl leukotriene receptor 1 (hCYSLTR1) antagonism	>49.8 µM
Human cysteinyl leukotriene receptor 1 (hCYSLTR1) agonism	>49.8 µM
Human purinergic receptor P2Y_1_ (hP2RY_1_) antagonism	>49.8 µM
Human purinergic receptor P2Y_1_ (hP2RY_1_) agonism	>49.8 µM
Human purinergic receptor P2Y_2_ (hP2RY_2_) antagonism	>49.8 µM
Human purinergic receptor P2Y_2_ (hP2RY_2_) agonism	>49.8 µM
Human purinergic receptor P2Y_11_ (hP2RY_11_) antagonism	>49.8 µM
Human purinergic receptor P2Y_11_ (hP2RY_11_) agonism	>49.8 µM

Abbreviations used in Table 4: GPCR, G protein-coupled receptor.

Further profiling was done using the CEREP SafetyScreen87 panel (Eurofins). This is an industry-standard *in vitro* profiling panel designed to identify off-target drug activity early in preclinical development. It contains 87 molecular targets, including ion channels, nuclear receptors, enzymes and transporters, but also several G protein-coupled receptor, including adrenergic, dopamine, serotonin, opioid, histamine, muscarinic and chemokine receptors. The CEREP safety screen showed that RWT020016 (tested at a dose of 10 µM) displays no major off-target activities. Follow-on functional study at Eurofins for 4 receptors where RWT020016 showed >50% inhibition in the primary CEREP screen revealed low micromolar antagonistic activity at the 5-HT2A receptor (IC_50_ = 4 μM) and also low agonistic activities at the KOR (EC_50_ = 9.9 μM). Both agonistic and antagonistic activities at the other two receptors (AR, PPARγ) were >100 μM. These findings support RWT020016 as a potent and highly selective GPR17 antagonist with a favorable safety profile.

RWT020016 meets Lipinski’s Rule of Five and it’s *in vitro* ADME (absorption, distribution, metabolism, excretion) drug-like properties translated well into an orally active small molecule (Table 5) with ideal PK parameters to support a QD (*quaque die*; once daily) dosing regimen. Pharmacokinetic (PK) studies after single oral administration in C57BL/6 mice revealed that the compound (at 3 mg/kg) showed high bioavailability (%F = 91%), peak plasma concentrations (C_max_) exceeding 12’000 ng/mL, and a brain to plasma ratio > 0.05 (unbound), which meets the commonly accepted criteria for brain penetration in non-perfused brain tissue.

**Table 5:** RWT020016 Physicochemical properties and *in vivo* PK parameters after single-dose oral administration.

Physicochemical properties				
MW	<500g/mol			
H-bond donors	2			
H-bond acceptors	<10			
cLogP	<5			
PK Parameters				
Dose	***t***_**1/2**_	**T**_**max,brain**_	**C**_**max,brain**_	**AUC**_**0-24h,brain**_
0.3 mg/kg	8.3 h	1 h	39 ng/g	588 h·ng/g
1 mg/kg	6.9 h	2 h	205 ng/g	2408 h·ng/g

Abbreviations used in Table 5: AUC, area under the curve; cLogP, calculated LogP – measure of compound’s lipophilicity; C_max_, maximal concentration; T_max_, time to reach maximal concentration; t_1/2_, compound’s half-life; MW, molecular weight; PK, pharmacokinetic.

**Fig 5:**
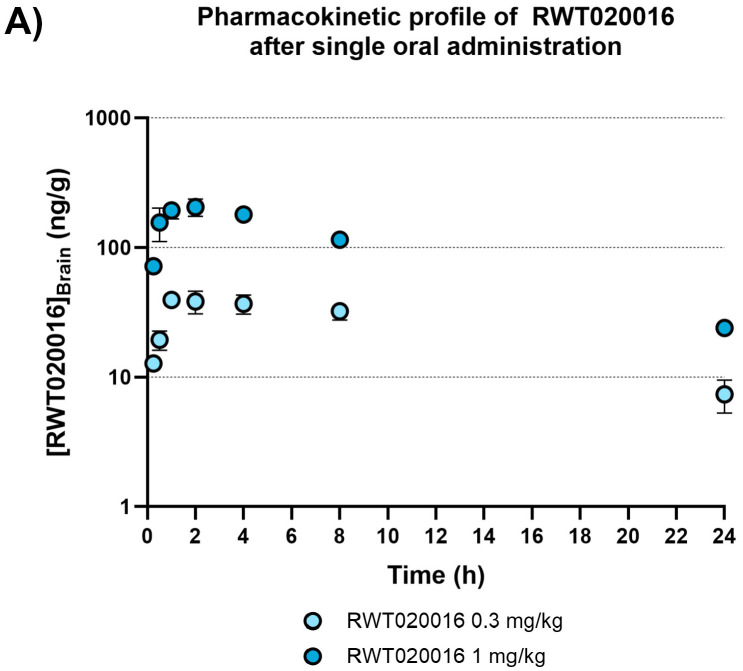
RWT020016 is a brain-penetrant GPR17 antagonist with dose-dependent activity. Pharmacokinetic profile of RWT020016 (0.3 and 1 mg/kg) and brain exposure time course (0.25, 0.5, 1, 2, 4, 8, 24h, N = 3 per time point) after single oral administration in mice. Data is presented as mean ± SD.

Overall, the compound distributed centrally with relevant total and dose-dependent brain exposures that elicited robust and reproducible pharmacodynamic effects (Fig 5). Importantly, highly similar total levels of RWT020016 were measured in the brain 2 h after dosing in our *in vivo* cuprizone experiments. The average for the 0.3 mg/kg dose was 56.6 ng/g (SD = 15.8); for the 1 mg/kg dose 232.2 ng/g (SD = 89.5).

### RWT020016 treatment results in robust functional remyelination after cuprizone exposure

**Fig 6:**
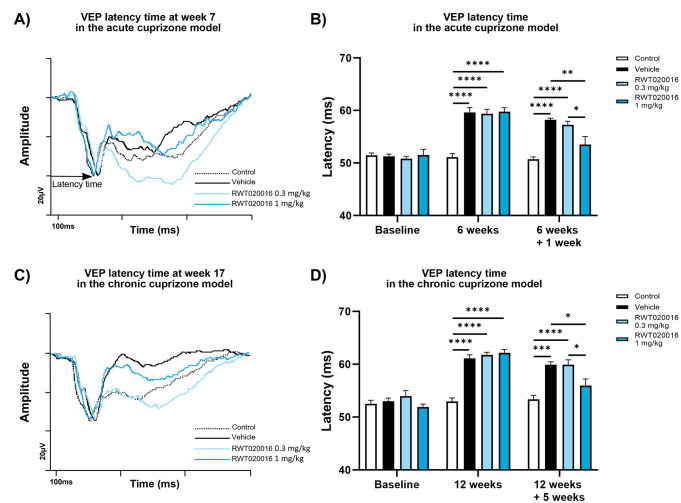
RWT020016 treatment improves VEP latency delays, indicating enhanced remyelination in cuprizone models. (A) Representative VEP traces recorded at week 7 from control, vehicle, and RWT020016-treated mice (0.3 mg/kg and 1 mg/kg) in the acute cuprizone model. Latency time is indicated on the graph. (B) Quantification of absolute VEP latency at week 7 in the acute model showed that both doses of RWT020016 improved VEP latency compared with vehicle, with a significant effect observed at 1 mg/kg (*p* = 0.003 vs. vehicle at week 7, One-way ANOVA with Dunnett’s multiple comparisons test per timepoint, N = 10-11). (C) Representative VEP traces recorded at week 17 in the chronic cuprizone model. (D) Quantification of absolute latency times in the chronic model showed a significant improvement in VEP latency time with RWT020016 treatment at 1 mg/kg compared with vehicle (*p* = 0.02 vs. vehicle at week 7, One-way ANOVA with Dunnett’s multiple comparisons test per timepoint, N = 10-11). Data are expressed as mean ± SEM. *p < 0.05, **p < 0.01, ***p < 0.001, ****p < 0.0001.

To determine whether RWT020016 treatment promotes functional recovery and remyelination, VEP latency was assessed following acute and chronic cuprizone-induced demyelination. In the acute model (Fig 6 A-B), the cuprizone-treated groups showed a significant delay in VEP latency of 8.4 ms (*p* < 0.0001 vs. control) after 6 weeks of cuprizone treatment, indicating impaired axonal signal conduction. One week after arresting the cuprizone treatment, the vehicle-treated animals showed spontaneous remyelination with a reduction of 1.4 ms, whereas RWT020016 treatment improved VEP latency in a dose-dependent manner compared with vehicle (1.0 ms reduction at 0.3 mg/kg and 4.7 ms reduction at 1 mg/kg RWT020016). Quantitative analysis confirmed a significant improvement in VEP latency in the 1 mg/kg group (*p* = 0.003 vs. vehicle; Fig 6 B), in line with the brain PK and brain exposure data and supporting the promyelinating activity of the compound. Similarly, in the chronic model (Fig 6 C-D), a delay in VEP latency of 8.1 ms was evident at week 12 of the cuprizone-treated groups, and a significantly increased recovery of VEP latency was observed at week 17 after 35 days of daily oral dosing of 1 mg/kg RWT020016 (4 ms reduction, *p* = 0.02 vs. vehicle) compared with vehicle-treated mice, indicating an endogenous latency improvement of 1.2 ms in vehicle-treated mice. In agreement with the lower brain exposure, limited improvement was seen in the 0.3 mg/kg RWT020016 group compared with vehicle-treated animals. Together, these findings demonstrate that RWT020016 accelerates spontaneous remyelination and improves functional outcomes in both acute and chronic cuprizone models.

**Fig 7:**
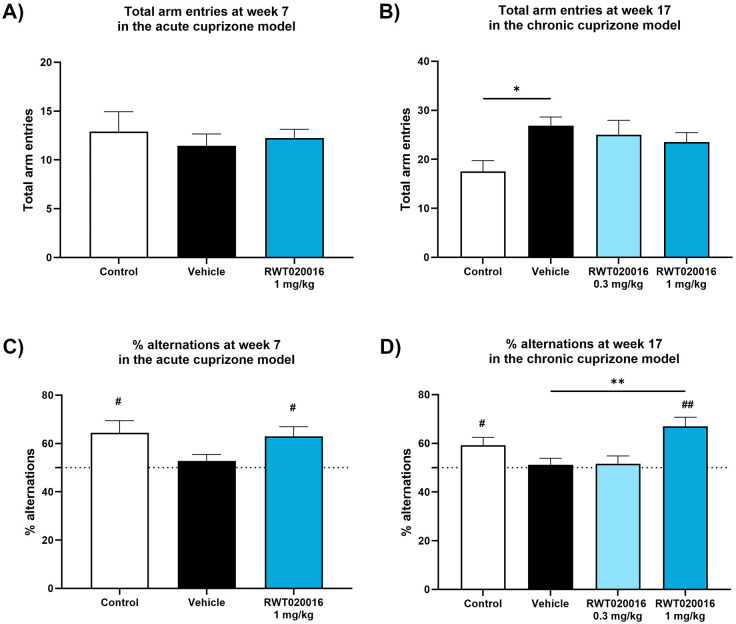
RWT020016 improves working memory performance in acute and chronic cuprizone models. (A) Total arm entries at week 7 in the acute cuprizone model show no significant differences between control, vehicle, and RWT020016-treated mice, indicating comparable locomotor activity. (B) Total arm entries at week 17 in the chronic cuprizone model increased in the cuprizone-treated groups with the vehicle group showing a significant increase in motor activity compared with control (*p* = 0.02 vs. control, One-way ANOVA with Dunnett’s multiple comparisons test, N = 9-11). (C) In the acute model, cuprizone treatment resulted in reduction of spontaneous alternation to the 50% chance level, as seen in the vehicle group at week 7. Treatment with RWT020016 (1 mg/kg) resulted in a significant recovery and revealed a performance above the 50% alternation level, which was the same as seen in the control mice (*p* = 0.016 for control and *p* = 0.015 for RWT020016 vs. hypothetical 50% alternation using one-sample t-test, N = 9-11). (D) Percentage of spontaneous alternations at week 17 reveals a cuprizone-induced impairment in working memory in the vehicle group. While the treatment with 0.3 mg/kg RWT020016 did not reveal any improvement, the RWT020016-treated mice at 1 mg/kg showed a significant recovery (*p* = 0.02 for control and *p* = 0.002 for 1 mg/kg RWT020016 vs. hypothetical 50% alternation using one-sample t-test, N = 9-11). In addition, RWT020016 (1 mg/kg) treatment showed increased percentage of alternations compared with vehicle (p = 0.004 vs. vehicle, One-way ANOVA with Dunnett’s multiple comparisons test, N = 9-11). Data are presented as mean ± SEM. *p < 0.05, **p < 0.01, #*p* < 0.05, ## *p* < 0.01. The dotted line (C,D) indicates chance performance (50%).

In addition, we assessed the impact of RWT020016 on spatial working memory in both cuprizone models. In the acute model at week 7, total arm entries were comparable across control, vehicle, and the RWT020016-treated groups, indicating no significant differences in general locomotor activity (Fig 7 A). However, the vehicle group exhibited a marked reduction in spontaneous alternation performance, reaching the chance level of 50%, whereas RWT020016-treated mice (1 mg/kg) demonstrated spontaneous alternations significantly above the level of 50% (*p* = 0.015), and performing at levels comparable with the control mice (Fig 7 C), suggesting restoration of the cuprizone-induced working memory deficit. In the chronic model at week 17, all cuprizone-treated mice showed increased total arm entries, with the vehicle group displaying a statistically significant elevation compared with controls (*p* = 0.02 vs. control, Fig 7 B), indicating sustained hyperactivity due to chronic cuprizone treatment. With regard to working memory performance, both the vehicle and RWT020016 0.3 mg/kg-treated groups failed to exceed the 50% alternation threshold, indicating impaired working memory capacity. Conversely, RWT020016 at 1 mg/kg significantly improved spontaneous alternation behavior relative to vehicle (*p* = 0.004 vs. vehicle) and was the only treatment group to perform significantly above the 50% chance level (Fig 7 D). Collectively, these findings demonstrate that RWT020016 effectively mitigates cuprizone-induced deficits in spatial working memory in both the acute and the chronic cuprizone demyelination model.

### RWT020016 treatment results in structural remyelination after cuprizone exposure

**Fig 8:**
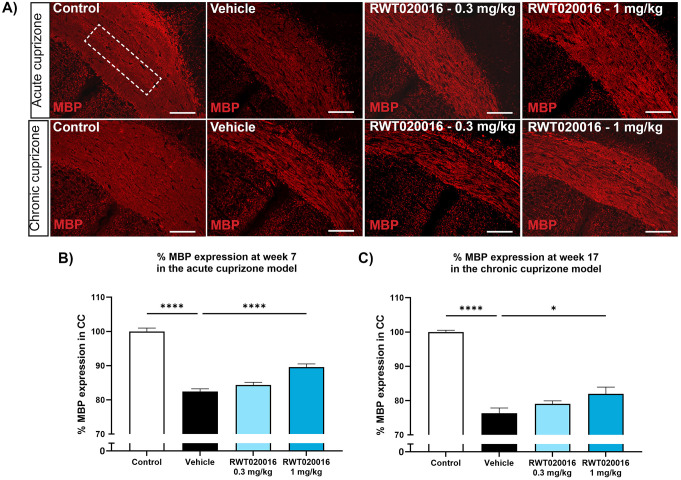
RWT020016 promotes remyelination in the corpus callosum in the acute and chronic cuprizone model. (A) Representative images of myelin basic protein (MBP) immunofluorescence in the CC at week 7 (acute model, top row) and week 17 (chronic model, bottom row). The dotted-line square in the acute control panel indicates the region of CC used for quantitative analysis. Demyelination was evident in vehicle-treated mice, with reduced MBP staining compared with controls. RWT020016 treatment at both 0.3 mg/kg and 1 mg/kg increased MBP protein expression. (B) Quantification of MBP protein expression in the acute model showed a significant reduction in the vehicle group compared with control (*p* < 0.0001 vs. vehicle, One-way ANOVA with Dunnett’s multiple comparisons test, N = 10-11), which was significantly elevated by RWT020016 at 1 mg/kg (*p* < 0.0001 vs. vehicle, One-way ANOVA with Dunnett’s multiple comparisons test, N = 10-11). (C) In the chronic model, MBP protein expression remained significantly reduced in the vehicle group compared with control (*p* < 0.0001 vs. vehicle, One-way ANOVA with Dunnett’s multiple comparisons test, N = 10-11) and was again significantly elevated by RWT020016 at 1 mg/kg (*p* = 0.01 vs. vehicle, One-way ANOVA with Dunnett’s multiple comparisons test, N = 10-11). Data are presented as mean ± SEM and are relative to the control mean (mean control values are set to 100%). *p < 0.05, ****p < 0.0001. Scale bars: 100 µm (A).

To evaluate the effect of RWT020016 on myelin integrity following cuprizone-induced demyelination, MBP expression in the CC was assessed by immunofluorescence staining for MBP at week 7 (acute model) and week 17 (chronic model). In the acute model, vehicle-treated mice exhibited substantial demyelination compared with controls, as evidenced by a marked reduction in MBP signal intensity (Fig 8 A, top row). Quantitative analysis confirmed a significant decrease in MBP protein expression in the vehicle group (*p* < 0.0001 vs. control), which was partially restored in the 0.3 mg/kg group and significantly improved at 1 mg/kg (*p* < 0.0001 vs. vehicle, Fig 8 B), indicating a dose-dependent remyelinating effect. A similar pattern was observed in the chronic model (Fig 8 A, bottom row). Chronic cuprizone exposure led to a sustained MBP loss in the vehicle-treated mice (*p* < 0.0001 vs. control). Treatment with RWT020016 at 1 mg/kg significantly enhanced MBP expression compared with vehicle (*p* = 0.01 vs. vehicle, Fig 8 C), while RWT020016 at 0.3 mg/kg produced a modest but statistically non-significant increase. These findings suggest that inhibiting GPR17 functions by RWT020016 promotes myelin protein expression and structural remyelination in the CC in both the acute and chronic model of cuprizone-induced demyelination.

### RWT020016 treatment reduces pathological abnormalities in the optic nerve after cuprizone treatment

**Fig 9:**
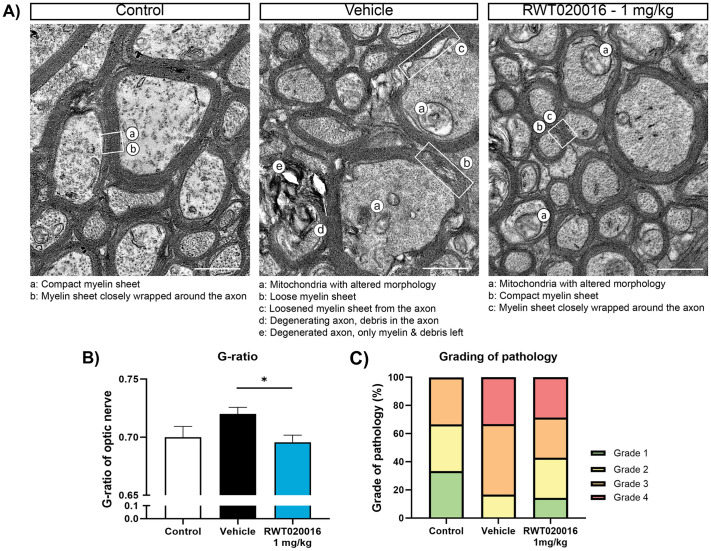
Transmission electron microscopy analysis of optic nerve and semi-quantitative analysis of pathology following RWT020016 treatment. (A) Representative images of the optic nerve from control (left), vehicle (middle) and RWT020016-treated mice (1 mg/kg, right). Pathological features are annotated in the images. (B) G-ratios of 125 axons per optic nerve, quantified from five TEM images per optic nerve section, were measured and showed a significant reduction following RWT020016 treatment as compared to vehicle (*p* = 0.049, One-way ANOVA with Dunnett’s multiple comparisons test, N = 6). (C) Semi-quantitative analysis involving scoring of optic nerve pathology of each group, representing the proportion of none (grade 1), mild (grade 2), moderate (grade 3) and severe (grade 4) pathology (N = 6-7). The comprehensive ultrastructural analysis showed that RWT020016 treatment reduced the proportion of moderate/severe pathology compared with the vehicle group, suggesting a beneficial effect on myelin integrity in the optic nerve. Data are presented as mean ± SEM. *p < 0.05. Scale bars: 1 µm (A).

To complement the functional remyelination data obtained via VEP in the optic nerve, a semi-quantitative TEM analysis was performed to assess structural changes in the chronic cuprizone model. We specifically focused on the chronic variant, as it more closely reflects the prolonged demyelination observed in human disease. Analysis of the g‑ratio revealed a trend toward increased values in mice exposed to cuprizone + vehicle compared to naïve controls, consistent with reduced/lost myelin thickness. Although this difference did not reach statistical significance, the direction of the effect, together with the heterogeneous nature of demyelination in the cuprizone model, suggests that these findings are more likely reflective of partial or incomplete myelin loss rather than a complete absence of structural pathology, as well as linked to the small sample size included in EM-analyses. At week 17, RWT020016 treatment significantly decreased the G-ratio (*p* = 0.049 vs. vehicle) in the optic nerve, indicating restoration of the myelin sheath (Fig 9 B). Furthermore, our qualitative investigation of the morphological features of the optic nerves revealed that the vehicle-treated group exhibited a substantial increase in the proportion of optic nerves displaying moderate to severe pathology (Grades 3–4) compared with the control group (Fig 9 A-C). These pathological alterations following cuprizone treatment included mitochondria with altered morphology, loose or disintegrating myelin, and degenerating axons (Fig 9 A, middle panel). In contrast, detailed assessments of these pathological features revealed substantial improvements in the RWT020016 group receiving 1 mg/kg relative to vehicle, particularly in reducing the number of axons with loose or detached myelin sheaths (Fig 9 A, right panel). Overall, treatment with RWT020016 at 1 mg/kg markedly reduced the proportion of optic nerves with high-grade pathology (Fig 9 B-C), highlighting the reparative effect of GPR17 antagonism on myelin integrity. Altogether, our TEM analysis confirms the functional and histological findings, highlighting that blocking GPR17 functions promotes myelin repair at the ultrastructural level.

## Discussion

Our study provides novel scientific evidence for the high therapeutic potential of targeting GPR17 for remyelination through pharmacological intervention. We demonstrate, for the first time, that RWT020016, an orally active, potent, selective and specific GPR17 antagonist, effectively enhances both structural and functional remyelination *in vivo* in a pathological context where GPR17 is dysregulated. PK and brain exposure data support a brain-specific effect of RWT020016 with target concentrations allowing effective blockade at a dose of 1 mg/kg. These findings are reinforced by concordant human transcriptomic and immunohistochemical data, as well as ultrastructural and behavioral evidence in rodent demyelination models.

GPR17, localized on the extracellular membrane of pre-myelinating cells, is an accessible target for pharmacological modulation [26]. Unlike earlier non-selective antagonists (e.g., Pranlukast, Montelukast, Cangrelor, for recent review see [33]), RWT020016 offers improved selectivity over closely related receptors such as P2RY_1_, _2_ and _11_ or CysLT_1_. While newer GPR17 antagonists have been identified [32], they lack *in vivo* validation in animal models of demyelinating diseases. Our findings extend prior genetic and pharmacological studies, collectively supporting GPR17 inhibition as a strategy to promote remyelination. While the identity and biology of the endogenous GPR17 ligand remain unresolved and thus represent an important limitation for interpreting the precise mode of action, our data support a functional role for RWT020016 under demyelinating conditions. This effect may reflect either competitive antagonism against an endogenous ligand or inverse agonistic activity at the GPR17 receptor. We demonstrate that RWT020016, even at the low dose of 1 mg/kg, enhances structural and functional remyelination *in vivo* in cuprizone demyelination mouse models, corroborating results from GPR17 KO models [11,12,14,67,68]. While genetic models provide a strong rationale for GPR17 as a target for remyelination, our pharmacological approach further supports its potential for translational development. Importantly, the data presented here were generated in a mechanistic de- and remyelination model that does not recapitulate the full inflammatory complexity of many demyelinating diseases. As such, effective therapeutic strategies will require not only the promotion of remyelination but also concomitant targeting of the primary drivers of demyelination, such as inflammation.

By leveraging 5 publicly available single-nucleus RNA sequencing datasets derived from postmortem human brain, we confirmed GPR17’s selective expression in oligodendrocyte lineage cells highlighting the enrichment in COPs in the adult human brain. Importantly, no expression of GPR17 transcripts was detected in astrocytes, microglia, or neurons, confirming the selective localization in the oligodendroglial populations. These data are in line with murine RNA sequencing data [69] and provide a complementary view and support of the hypothesis put forward in the recent review by Fang et al. who propose that GPR17 expression marks a population of COPs that exit the proliferation cycle, which downregulate NG2, and proceed to differentiation into myelinating oligodendroglia [70]. Our data is also consistent with prior genetic and immunohistochemical findings in mice [12], as well as postmortem human brain tissue [21]. Importantly, our immunohistochemical data provides independent confirmation of the findings by Angelini et al, who used a custom-made antibody to analyze GPR17 in patients with MS and offers a more nuanced qualitative characterization of GPR17 protein localization. GPR17-positive COPs, identified through co-localization with BCAS1, exhibited a distinct spatial association with both myelin structures and immune cells. Across all the analyzed samples and lesions, GPR17 expression was predominantly detected in active or chronically demyelinated, non-remyelinated lesions, whereas it was absent in shadow plaques, which are indicative of partial remyelination. Although these observations are descriptive in nature, they should be interpreted in the context of the inherent limitations of postmortem human tissue analysis. The relatively small number of available samples, combined with substantial inter-patient heterogeneity, variability in lesion size and stage, and differences in tissue preservation and processing, limit the robustness and interpretability of formal quantitative comparisons across lesion types. Despite these limitations, the observed spatial patterns are consistent with a model in which sustained GPR17 expression is associated with impaired committed oligodendrocyte precursor (COP) differentiation. In this context, the absence of GPR17 immunoreactivity in remyelinated lesions supports the hypothesis that downregulation of GPR17 may be required to enable effective oligodendrocyte maturation and remyelination, although causality cannot be inferred from these descriptive observations alone.

Our data supports a stage-specific effect of GPR17 antagonists on committed OPC differentiation *in vivo*; however, the downstream molecular mechanisms remain to be further elucidated. Our screening assays used cAMP and Ca^2+^ levels as readout for downstream signaling events mediated by Gαi/o and Gαq proteins, with revealed higher potencies of RWT020016 measured in the human Gαi/o-mediated cAMP assay as compared to the Ca^2+^ assay, while the murine assays showed slightly higher potencies in the Ca^2+^ assay (Table 3). The data is in line with previous *in vitro* studies showing that GPR17 signaling is coupled to Gαi/o proteins and negatively regulates intracellular cAMP levels, influencing pathways such as cAMP/PKA and MAPK/ERK signaling—both known modulators of oligodendrocyte differentiation [11,12,71]. In addition, transcription factors such as SOX10 and MYRF act as central regulators of myelin gene expression and oligodendrocyte maturation [72,73]. While these studies provide fundamental insights into GPR17 function in OPC differentiation, the effects of pharmacological inhibition using selective GPR17 antagonists or inverse agonists on downstream signaling events need further investigations. An important unresolved issue here concerns the unknown or debated identity of GPR17’s endogenous ligand. This is particularly relevant because demyelinating insults are expected to release pathology-associated signals that upregulate GPR17 and may act as endogenous ligands. Future studies combining *in vitro* OPC differentiation models that mimic pathological conditions with pathway-specific and transcriptional analyses will be essential to clarify the precise mode of action of GPR17 antagonism and the mechanisms underlying the differentiation block at the committed OPC stage. Consistent with the human data, GPR17 expression was strongly upregulated in the corpus callosum following cuprizone-induced demyelination, in line with previous findings [15], supporting its role in endogenous myelin repair and regeneration after brain injury. The oligodendrocyte toxicity and subsequent demyelination is also accompanied by microglial activation—a critical process for clearing cellular debris. While the present study focused on GPR17 expression and function within the oligodendroglial lineage, previous work has shown that GPR17 can be induced in microglia/macrophages under certain pathological conditions, such as after ischemic CNS injury in mice, where it may contribute to damage sensing and repair responses [11]. Importantly, however, studies in the cuprizone model did not detect substantial GPR17 expression in Iba1-positive microglia/macrophages, suggesting that GPR17 signaling in this context is predominantly oligodendroglial [13]. In line, our postmortem human analysis did not reveal an overlap between anti-HLA (marker for myeloid cells, including microglia) and anti-GPR17 immunoreactivity. The exclusive expression of GPR17 in OPCs is further shown in our bioinformatic analysis of human scRNAseq data, where transcripts were absent in neurons and other glial populations (S1 Fig). Nevertheless, indirect effects of GPR17 antagonism on the inflammatory milieu cannot be fully excluded, and future studies employing cell-type–specific expression analyses and inflammatory profiling will be valuable to further clarify potential influence on microglial functions.

These immunohistochemical findings are further substantiated by our electrophysiological assessment using VEP recordings, an emerging translatable biomarker of demyelination and remyelination, both in preclinical models [65,74,75] and clinically in MS patients [38,76]. We demonstrated that the cuprizone diet induced a robust delay in VEP latency without affecting amplitude, indicating demyelination without overt retinal ganglion cell loss. Repeated daily dosing of RWT020016 significantly improved VEP latency in both demyelination mouse models, strongly supporting its remyelination-promoting effect. As the electrophysiological recordings represent a functional surrogate marker of changes along the optic pathway, VEP findings alone cannot definitively distinguish remyelination from other contributors to improved conduction. Accordingly, while these data are consistent with enhanced myelin repair, we cannot exclude that compensatory conduction mechanisms or improved axonal preservation may also have contributed to the observed effects. Importantly, the interpretation of remyelination is based on the convergence of electrophysiological, histological, and ultrastructural findings rather than VEP data alone. Behavioral tests in the cuprizone model also revealed a recovery in spatial working memory performance following GPR17 antagonism via oral dosing of RWT020016 without affecting locomotion. In the present study, locomotor activity was assessed through total arm entries, but additional behavioral controls would further strengthen interpretation, as the spontaneous alternation performance can be influenced by factors beyond working memory alone, including also anxiety and exploratory behaviour. Accordingly, our findings should be interpreted as supportive, rather than definitive, evidence of functional recovery. Addressing these additional factors will be an important direction in future studies. Nevertheless, these data suggest that promoting remyelination via GPR17 antagonists might also provide cognitive benefits. This is consistent with previous findings reporting neuroprotective effects of GPR17 antagonism against LPS-induced cognitive deficits [68]. These findings are also clinically relevant as MS patients frequently present with cognitive symptoms, including slowed processing speed and working memory deficits [66,77], and highlight the therapeutic potential of GPR17 antagonists to improve cognitive performance in MS.

Structural improvements following RWT020016 treatment were confirmed by the elevated MBP protein levels in the corpus callosum, as well as in the optic nerve at the ultrastructural level in the cuprizone mouse models. Conducting a G-ratio analysis, a well-established method to investigate myelin thickness [78], as well as a comprehensive qualitative structural analysis with a pathological scoring integrating mitochondrial morphology, axonal and myelin integrity, we selected robust and biologically relevant endpoints to assess axonal repair mechanisms including remyelination. While larger sample sizes will be required for formal statistical comparisons at the ultrastructural level, our investigations nevertheless indicate that RWT020016-treated mice exhibited a significant restoration of g-ratios as well as markedly improved axonal integrity and myelin restoration relative to the vehicle group, which displayed pronounced myelin disruption and mitochondrial abnormalities. While an expanded morphometric analysis will further strengthen our data, the findings are consistent with a remyelination-promoting effect and support the relevance of the cuprizone model for capturing disruptions in axon–myelin coupling that may contribute to disease progression in MS.

While our data supports a strong link between structural and functional recovery following oral RWT020016 treatment, we acknowledge that additional investigations are needed to further validate and extend our findings in future studies. One limitation of our study is the focus on male mice only, which was chosen to reduce biological variability and increase statistical power in this initial pharmacological evaluation. However, this limits the generalizability of the findings, and future studies will specifically include both sexes to assess potential sex-dependent differences in pharmacokinetics, target engagement, and remyelination efficacy. Additional ultrastructural investigations in the corpus callosum – a white matter structure strongly affected by the cuprizone treatment – will substantiate the myelin basic protein data. Moreover, immunohistochemical and volumetric cellular density analyses using markers of mature oligodendrocytes will provide additional confirmation that blocking GPR17 functions promotes differentiation of COPs to myelinating oligodendroglia. Such investigations will also be relevant to address putative contributions from surviving mature oligodendrocytes to remyelination. While OPC- and mature oligodendrocyte–mediated processes likely operate in parallel, current evidence indicates that efficient remyelination is driven predominantly by newly generated oligodendrocytes, with only a limited and context-dependent contribution from pre-existing cells [79]. Accordingly, GPR17 antagonism primarily targets the OPC-mediated axis of repair, although indirect effects on oligodendrocyte metabolism [80] cannot be entirely excluded and may further support remyelination. Histology of optic nerves could also strengthen our findings by expanding the analysis to larger parts as compared to the limited area included in ultrastructural investigations.

Another important focus of future studies will be the demonstration of drug target engagement. Although quantifying GPR17 ⁺ cell density might appear to address this, it is not an optimal measure for this receptor. GPR17 expression is not known to undergo autoregulatory feedback in response to pharmacological inhibition, and there is currently no evidence that exposure to a GPR17 antagonist or inverse agonist alters the number of GPR17 ⁺ oligodendroglial cells. Therefore, direct assessment of downstream signaling events in GPR17‑expressing cells will provide a more informative and reliable readout of target engagement.

Although several therapeutic remyelination strategies, including Bexarotene, Opicinumab, and Clemastine, have encountered challenges in clinical testing that were either linked to target selectivity, efficacy or adverse safety profiles, they have provided important insights for the development of future remyelinating therapies [81–83]. In this context, selective GPR17 antagonists, such as RWT020016 and optimized analogues, demonstrate better brain-specific activity and may offer a safety advantage given the selective expression of GPR17 in COPs. Assessing long-term safety will remain a critical priority—not only for patients with MS but also for other chronic CNS diseases where oligodendroglial dysfunction is increasingly recognized. Although GPR17 knockout mice do not exhibit overt abnormalities, the chronic safety profile of GPR17 antagonists has yet to be established. A definitive demonstration of *in* *vivo* target specificity would benefit from studies in global or conditional GPR17 knockout models. Dedicated studies including genetic validation, as well as evaluating long-term dosing, toxicology, and potential off-target effects *in vivo* will therefore be essential to support clinical translation. Given the dynamic nature of GPR17 expression within the OPC population, sustained blockade may not be required. Exploring different compound properties and dosing intervals could provide valuable insights into optimal treatment regimens and will remain an important area of future investigations.

## Conclusions

**Fig 10:**
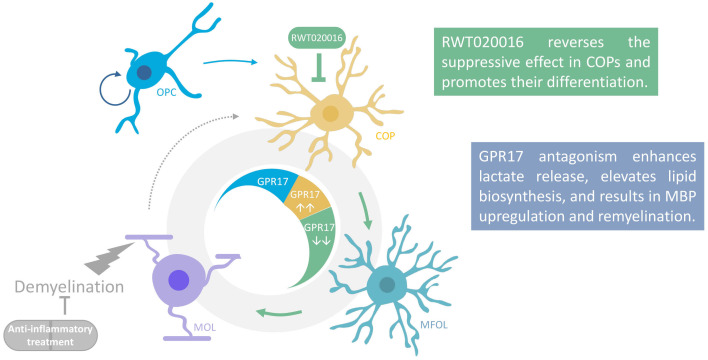
Proposed mechanism for RWT020016 in promoting remyelination following demyelinating insults in the brain. Under demyelinating conditions, oligodendrocyte precursor cells (OPCs) are recruited to the sites of lesions, starting to express GPR17 and differentiate into committed OPCs (COPs). Demyelinating conditions are associated with chronic upregulation of GPR17, leading to a differentiation arrest of COPs. RWT020016 inhibits GPR17 signaling, releasing the differentiation block and allowing the cells to progress to mature myelin-forming oligodendrocytes (MFOLs). Furthermore, GPR17 inhibition is thought to promote oligodendrocyte differentiation and remyelination, potentially via enhanced lactate release, increased lipid biosynthesis, and elevated MBP expression [9,26,33,80].

Our studies combining VEP, behavioural assessment, histology and ultrastructural analysis of myelin repair revealed that RWT020016 promotes remyelination likely via OPC maturation by effectively and selectively inhibiting GPR17, thereby overcoming the differentiation block and promoting myelin protein synthesis. Previous reports also provided evidence that blocking GPR17 functions changes activity of several genes involved in glucose metabolism and lipid biosynthesis [9,80]. The activation of lipid biosynthesis was associated with an increase in lactate release, hypothesized to provide metabolic support to neurons [9,80], which can ultimately lead to structural and functional remyelination (Fig 10). These findings position GPR17 inhibition as a promising and clinically relevant strategy for treating demyelinating diseases.

## Supporting information

S1 Fig UMAP visualization and cell type marker expression in single-nucleus RNA sequencing data. (A) UMAP plot of integrated datasets showing clustering of glial cells from five different datasets (color-coded by dataset). (B) UMAP plot annotated by cell type: astrocytes, oligodendrocytes (olig), and microglia. (C-V) UMAP plots showing the expression of canonical marker genes. The intensity of the violet color indicates the expression level of each gene in individual cells. Color scale represents gene expression levels (log-normalized counts). Gray indicates low or no expression. (FYB1, FYN binding protein 1; C3, Complement component 3; AQP4, Aquaporin 4; GJA1, Gap junction protein alpha 1; MOBP, myelin-associated oligodendrocyte basic protein; MBP, myelin basic protein; GRIN1, Glutamate ionotropic receptor NMDA type subunit 1; GAD2, Glutamate decarboxylase 2; SLC17A7, Solute carrier family 17 member 7; CLDN5, Claudin 5; SOX6, SRY-Box transcription factor 6; BMP4, bone morphogenetic protein 4; TNS3, Tensin 3, FYN, Src family tyrosine kinase; TCF7L2, Transcription factor 7 like 2; CASR, Calcium sensing receptor; ITPR2, Inositol 1,4,5-triphosphate receptor type 2).
(JPG)
