# Supplementary figures and images for: Selective GPR17 antagonism enhances structural and functional recovery in animal models of demyelination
Source: PLoS One. 2026 Jul 27;21(7):e0354525. doi: 10.1371/journal.pone.0354525 (PMC13405111; doi:10.1371/journal.pone.0354525)

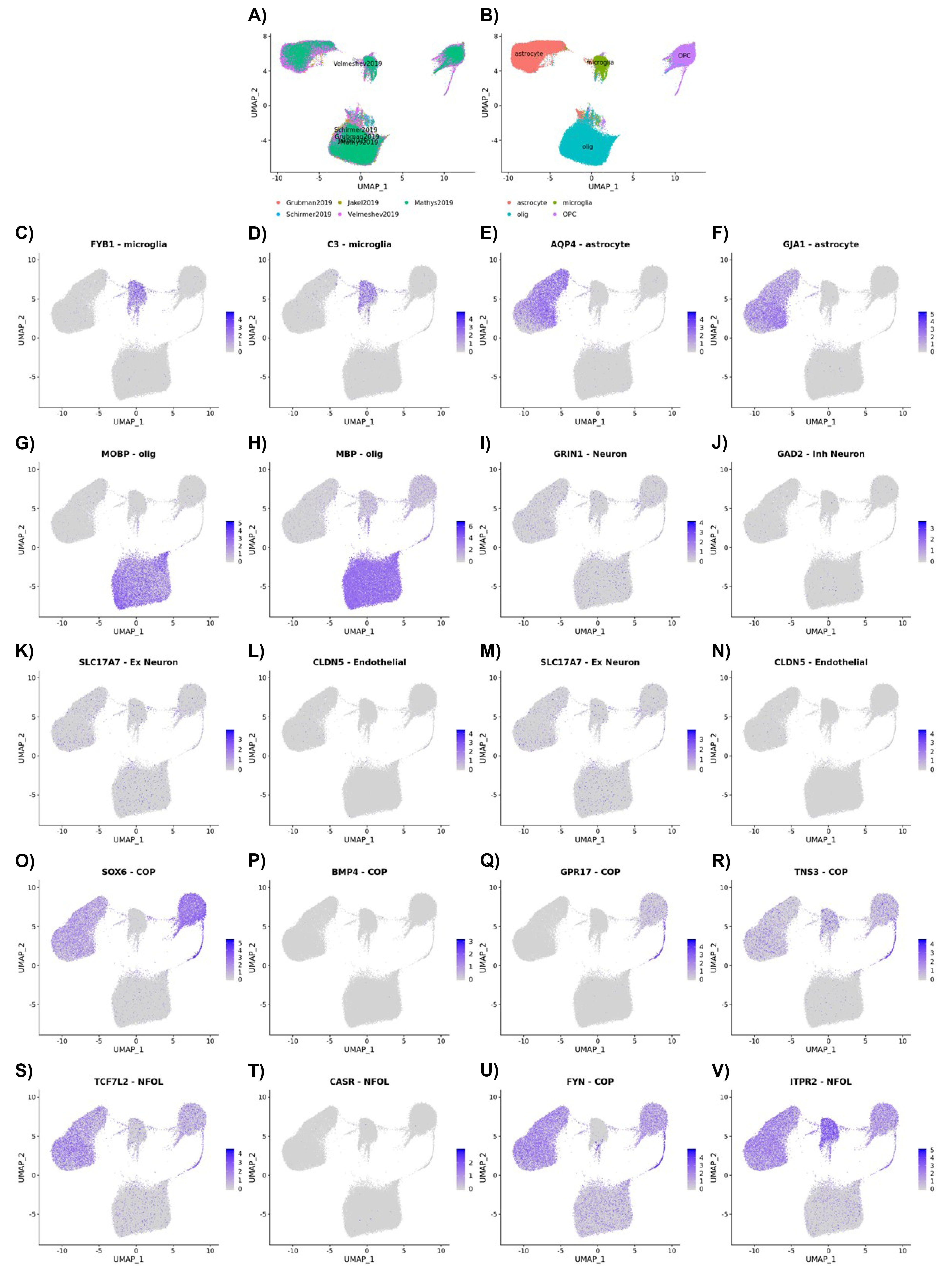

Supplement: S1 Fig — (A) UMAP plot of integrated datasets showing clustering of glial cells from five different datasets (color-coded by dataset). (B) UMAP plot annotated by cell type: astrocytes, oligodendrocytes (olig), and microglia. (C-V) UMAP plots showing the expression of canonical marker genes. The intensity of the violet color indicates the expression level of each gene in individual cells. Color scale represents gene expression levels (log-normalized counts). Gray indicates low or no expression. (FYB1, FYN binding protein 1; C3, Complement component 3; AQP4, Aquaporin 4; GJA1, Gap junction protein alpha 1; MOBP, myelin-associated oligodendrocyte basic protein; MBP, myelin basic protein; GRIN1, Glutamate ionotropic receptor NMDA type subunit 1; GAD2, Glutamate decarboxylase 2; SLC17A7, Solute carrier family 17 member 7; CLDN5, Claudin 5; SOX6, SRY-Box transcription factor 6; BMP4, bone morphogenetic protein 4; TNS3, Tensin 3, FYN, Src family tyrosine kinase; TCF7L2, Transcription factor 7 like 2; CASR, Calcium sensing receptor; ITPR2, Inositol 1,4,5-triphosphate receptor type 2). (JPG) [file pone.0354525.s001.jpg]
